# Assessing Prevalence and Characteristics of Oro-bulbar Involvement in Children and Adults with SMA Type 2 and 3 Using a Multimodal Approach

**DOI:** 10.1007/s00455-023-10584-z

**Published:** 2023-06-08

**Authors:** Federica Trucco, Francesca Salmin, Andrea Lizio, Giorgia Coratti, Emilio Albamonte, Maria Chiara Frisoni, Luca Mauro, Elena Carraro, Giovanni Palazzo, Jessica Lops, Camilla Cattaneo, Susanna Pozzi, Jacopo Casiraghi, Alessandra Di Bari, Beatrice Berti, Giulia Stanca, Martina Ricci, Marika Pane, Chad Heatwole, Nuran Dilek, Eugenio Mercuri, Valeria Ada Sansone

**Affiliations:** 1 Neurorehabilitation Unit, The NeMo Clinical Center in Milan, University of Milan, ASST Niguarda, Piazza Ospedale Maggiore, 3, 20162 Milan, Italy; 2 Dubowitz Neuromuscular Centre, UCL Great Ormond Street Institute of Child Health and Great Ormond Street Hospital, London, UK; 3 Dept Paediatric Respiratory Medicine, Royal Brompton Hospital, London, UK; 4 Centro Clinico Nemo, Fondazione Serena Onlus, Milan, Italy; 5 Pediatric Neurology, Università Cattolica del Sacro Cuore, Rome, Italy; 6 Centro Clinico Nemo, Fondazione Policlinico Universitario Agostino Gemelli IRCCS, Rome, Italy; 7 Center for Health and Technology (CHeT), The University of Rochester Medical Center, Rochester, NY USA

**Keywords:** Spinal muscular atrophy, Oro-bulbar involvement, Mastication, Swallowing, Nusinersen

## Abstract

Bulbar and jaw muscles are impaired in patients with Spinal Muscular Atrophy (SMA) but the assessment of their severity and progression are limited by the lack of age-appropriate and disease-specific measures. We investigated mastication and swallowing in children and adults with SMA, sitters and walkers. In a 2-year multicentre cross-sectional prospective study, lip and tongue strength (Iowa Oral Performance Instrument), chewing and swallowing (Test of Masticating and Swallowing Solids), active mouth opening (aMMO) were compared to age-appropriate normative data. The perceived burden of oro-bulbar involvement (SMA-Health Index) was recorded. 78 patients were included, 45 children (median age 7.4 years),22 adults (median age 26.8 years) on nusinersen and 11 untreated (median age 32.7 years). Forty-three percent children had reduced mouth opening, 50% had prolonged total time to eat. These issues were more prominent in sitters than in walkers (*p* = 0.019, *p* = 0.014). Sixty-six percent needed increased swallows for bolus clearance. Nusinersen treated adults had median aMMO, tongue strength and total time at TOMASS values within normal range (*z* score: −1.40, −1.22, −1.32, respectively) whereas untreated adults had reduced aMMO (*z* score: −2.68) and tongue strength (*z* score: −2.20). Only a minority of children (2/17) and treated adults (5/21) reported burden in swallowing or mastication compared to all untreated adults (5/5). After 16 months, mastication and swallowing were stable in treated children and adults, whether sitters or walkers. The reported multimodal approach to assess oro-bulbar functions demonstrate that swallowing and mastication are impaired in SMA despite patients’ perception. These results suggest a trend towards stabilization of oro-bulbar function in patients on long-term treatment with nusinersen.

**Supplementary Information:**

The online version contains supplementary material available at 10.1007/s00455-023-10584-z.

## Introduction

Spinal Muscular Atrophy (SMA) is a neuromuscular disease characterized by the degeneration of the anterior motor neurons. It is caused by the deletion of the Survival Motor Neuron (*SMN1*) gene on chromosome 5 resulting in the production of a truncated non-functional survival motor neuron protein [1, 2]. The *SMN* locus contains a paralogue gene, *SMN2,* which undergoes alternative splicing and produces a truncated mRNA isoform. Only about 10% of *SMN2* pre-mRNA is properly spliced and subsequently translated into full-length SMN protein [3].

The motor neuron degeneration results in patients with SMA either never acquiring (SMA type 1) or losing muscle bulk and strength over time, with the truncal and the proximal limb muscles being the first and most severely affected (SMA type 2–4) [4–8]. As brainstem innervated muscles are also impaired in SMA, oro-bulbar issues are commonly described.

Mastication and swallowing difficulties, assessed by the Test of Mastication and Swallowing solids (TOMASS), are particularly affected in these patients. They result from the complex interplay between the weakness of jaw and submental muscles and the anatomical and mechanical limitations causing the reduction of mouth opening and ineffective tongue movements that ultimately impair the ability to clear food in the mouth [9–13]. In addition, postural abnormalities (e.g., head tilt) in patients with SMA further affect mastication and swallowing either favoring penetration, as previously described, or representing a compensatory mechanism [10, 14–16].

The prevalence and characteristics of oro-bulbar function in paediatric and adult patients with SMA, and the relationship between mastication, oral and pharyngeal phase of swallowing have not been completely defined. Whilst the mandibular range of motion and the mastication and swallowing abilities have already been assessed in type 2 SMA via the measurement of Active maximal mouth opening (aMMO) [14, 17–19] and the TOMASS [15, 20], the objective assessment of lip and tongue strength via the Iowa Oral Performance Instrument (IOPI) has been used in some muscle disorders [21] but not in SMA. Also, despite a significant effort to identify the most specific patient-reported outcome measure (PROM) for mastication and swallowing in SMA patients [13, 22, 23], there is still no agreement on one specific scale. The SMA-Health Index (SMA-HI) is a SMA-tailored and validated PROM which assesses, amongst other disease-burdening issues, the impact of oro-bulbar dysfunction on the daily life of patients. [24]

The main aim of this study was to explore the prevalence and characteristics of mastication and swallowing issues in a cohort of paediatric and adult patients living with SMA types 2 and 3 using a set of combined objective measures and subjective reports. Because at the time of enrolment for this study, nusinersen was the only drug available for types 2 and 3 SMA patients with a high prevalence of treated patients we also aimed to describe, as an exploratory endpoint, the longitudinal progression of oro-bulbar in treated subjects.

## Patients and Methods

This is a two-year (2019–2020) multicentre prospective cross-sectional study conducted at two Italian highly specialized neuromuscular sites (The NeMo Clinical Centers—a. Dept Neurorehabilitation, University of Milan and b. Dept Pediatric Neurology, Università Cattolica del Sacro Cuore, Rome) investigating swallowing and mastication in patients with SMA type 2 and 3.

Neuromotor data were collected as part of an academic disease registry (Italian arm of the international SMA consortium, iSMAc), while oro-bulbar function data were collected in a specifically built database in RedCap. All data were collected after patient consent and IRB approval (n. 290-2705220).

### Patient Population

Patients with a genetically confirmed diagnosis of 5q-Spinal Muscular Atrophy were stratified, based on their functional status, as sitters (defined as score of 2 on the first item of the HFMSE) and walkers (defined as able to walk 10 m independently).

For each patient anthropometrics and age of symptoms onset were recorded. In sitters the recumbent or ulnar length (as per Gauld equation) was used as surrogate for height. [25] Body mass index (BMI) was compared to standard growth charts. In children the BMI was calculated using the center for Disease and Prevention Children’s BMI Tool (https://www.cdc.gov) and WHO reference curves (https://www.who.int/growthref/who2007_bmi_for_age/en/). Underweight was defined as a gender- and age-adjusted BMI < 5th percentile, whilst overweight was defined as an age-adjusted BMI > 85th percentile [16, 26]. In adults, a BMI < 18.5 m^2^/kg was considered as underweight and a BMI > 25 m^2^/kg was considered overweight as per WHO thresholds [27].

The study included both patients treated with nusinersen and those not receiving any treatment. At the time of the study nusinersen was the only commercially available treatment for type 2 and type 3 SMA. Patients untreated and those treated with 12 mg intrathecal nusinersen at maintenance regime (i.e., any visit after loading dose) were included. The age at first nusinersen administration was collected in treated patients.

Patients enrolled in interventional clinical trials as well as patients requiring invasive ventilation (e.g., tracheostomy) were excluded.

### Assessments of Oro-Bulbar Function

#### Lip and Tongue Strength

##### Iowa Oral Performance Instrument (IOPI) [28]

IOPI is a validated measure in patients affected by oropharyngeal muscular dystrophy and is currently being used in patients with Myotonic Dystrophies (TREAT-CDM at the University of Virginia, USA; and GUP19002, grant given to vs from Telethon and the Italian Muscular Dystrophy Association) [21, 28].

IOPI consists of a pressure bulb attached to a manometer. Lip strength was measured by squeezing or pursing the lips together while the examiner applied steady linear force to remove it. To measure tongue strength subjects were asked to push the bulb to the hard palate on the alveolar ridge just behind the upper central incisors for 3–5 s (repeated 3 times with a 30 s interval between trials). For each task, three attempts were conducted, and the maximum pressure generated was recorded [29] [30].

Lip strength, expressed in kPa, was only available for comparison in a population of healthy adults. [31] Tongue strength, expressed in kPa, was compared to age-appropriate values in healthy population. [31, 32]

#### Mastication and Swallowing

##### Active Maximum Mouth Opening (aMMO)

Participants were asked to open their mouth as wide as possible. The distance between the central incisal edges of the right upper and lower front teeth (minus the negative overbite) or from the upper and lower gum border was measured with a metal ruler [19, 33].

Values obtained were compared to age-appropriate published normative data [33, 34].

##### Test of Masticating and Swallowing Solids in Children (TOMASS-C) and Adults (TOMASS-A)

This is a validated tool for patients above 3 years of age measuring the efficiency of solid bolus intake by quantitative parameters namely number of bites, masticatory cycles (i.e., one cycle is defined as the opening and the closing of the jaw), swallows and the total time needed to finish the cracker.

Patients were required to ingest a cracker Gran Pavesi™ following the instruction “eat this as quickly as is comfortably possible and when you’re finished, say your name”. The test was videoed and timed.

Each of the four domains of TOMASS was compared to gender and age-appropriate normative data [20, 35]. A number of bites, masticatory cycles and swallows above normative values indicate the impairment of each ability whilst a prolonged time suggests a dysfunction in the whole process.

### Patient-Reported Outcome Measures (PROMs)

#### The Spinal Muscular Atrophy Health Index (SMA-HI), Italian Version

This scale has been developed as a patients’ reported disease burden outcome using FDA guidelines for SMA patients from 12 to 85 years of age. [36, 37]It has been recently translated and validated in Italian. [24] The higher the SMA-HI score, the worse is the patient perception of burden.

### Statistical Analysis

All variables were tested using Shapiro–Wilk test and Levene’s test to assess the normality of the distribution and the homogeneity of the variance, respectively.

Data were reported in text and tables as median and interquartile range for continuous variables, and frequency and percentage for categorical ones.

For the cross-sectional analysis, considering aMMO, IOPI and TOMASS separately, the first available evaluation per patient was considered.

Results of the objective measures in both paediatrics and adults’ patients were presented as z-scores. In detail, aMMO was standardized into z-scores using paediatrics’ Swiss [33] and adults’ American [38] normative data; IOPI values were standardized into z-scores using American normative data for both adults (lip and tongue strength) and paediatrics (tongue strength) patients, respectively [31, 32]; and number of bites, mastication cycles, swallows, and total time (in seconds) to eat the cracker were standardized into z-scores using normative data from Netherlands, Germany, New Zealand, Portugal and Italy’s children [20, 35]. *Z*-scores of < −1.96 (aMMO, IOPI) or >  + 1.96 (TOMASS) were considered abnormal.

The Mann–Whitney U test and the Fisher exact test were used to evaluate the differences between walkers and sitters’ patients considering, respectively, the z-scores and the prevalence of abnormal subjects, for each objective measure separately.

The score of the swallowing sub-domain of SMA-HI was transformed to a 0–100 scale by expressing it as a percentage of the maximum possible value. The subjective perception was considered as “no burdensome” if its score was equal to 0, with the score of 100 representing the most severe disease burden.

The relation between aMMO and time to eat the cracker assessed by TOMASS was explored using univariate linear regression, evaluating the statistical significance of aMMO’s contribution to the time needed to finish a standardized cracker, expressing the result in terms of percentage of decrease in time needed to finish the cracker for each mm of increasing in aMMO.

Finally, the progression of swallowing and mastication over time was showed using a line plot (spaghetti plot) of each considered objective measure, plotted for all the available time-points per patient. A mixed model for repeated measures was used to statistically evaluate the change over time in each objective measure, separately.

All tests were two-tailed, and a *p*-value < 0.05 was considered statistically significant. All statistical analyses were performed using SAS 9.3 (SAS Institute, Inc, Cary, NC) software.

## Results

### Study population

**Fig. 1:**
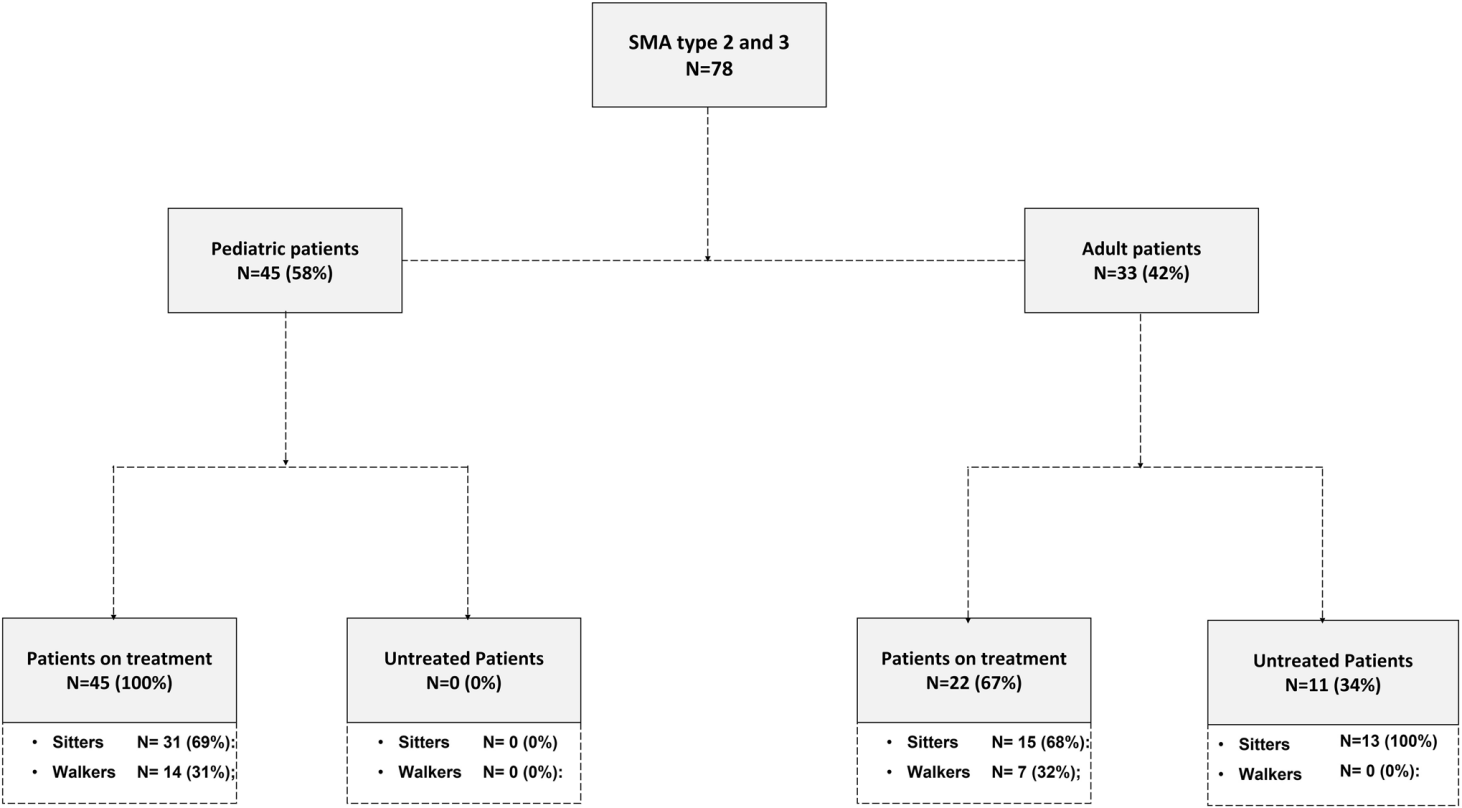
CONSORT diagram of the study population

Seventy-eight patients were included. Forty-five were children (31 sitters, 14 walkers), all on steady treatment with nusinersen, and 33 were adults, 22 on nusinersen (15 sitters, 7 walkers) and 11 untreated (all sitters) (Fig. 1).

At enrolment, the median age of the 45 children was 7.42 [5.69–9.69] years. All were able to eat solid food without dietary restriction. The median BMI was 15.67 [13.52–19.05] and 13 of 45 (29%) patients were underweight. Two patients (4%) had required gastrostomy due to previous findings of an unsafe swallowing at videofluoroscopy, with one of them still being underweight per age.

Of the 22 adult patients on nusinersen, 15 were functionally sitters and 7 walkers. Their median age was 26.80 [24.10–48.42] years. Four of the 22 (18%) had a BMI below 18 kg/m^2^.

Eleven adults were untreated at enrolment, all functionally sitters. Their median age was 32.67 [21.55–41.31] years. One patient (9%) had a BMI below 18 kg/m^2^. Two patients (18%) required an adapted (semi-solid) food texture (Table 1).

**Table 1:** Demographic and anthropometric features of the cohort of SMA patients at first evaluation

	Paediatric patients (*n* = 45)	Treated Adult (*n* = 22)	Untreated Adults (*n* = 11)
Age, median [IQR], y	7.42 [5.69–9.69]	26.80 [24.10–48.42]	32.67 [21.55–41.31]
BMI, median [IQR], kg/m2	15.67 [13.52–19.05]	22.88 [20.75–26.22]	22.23 [21.73–27.03]
Underweight	13 (28.89)	4 (18.18)	1 (14.29)
Normal BMI	17 (37.78)	10 (45.45)	4 (57.14)
Overweight	14 (31.11)	8 (36.36)	2 (28.57)
*Missing*	1 (%)	0 (%)	4 (%)
Ulna length, median [IQR], cm	18.00 [16.00–22.00]	25.50 [24.00–27.00]	26.00 [24.00–26.00]
Gender, n (%)			
Male	21 (46.67)	13 (59.09)	7 (63.64)
Female	24 (53.33)	9 (40.91)	4 (36.36)
SMA type, n (%)			
SMA 2	28 (62.22)	5 (22.73)	7 (63.64)
SMA 3	17 (37.78)	17 (77.27)	4 (36.36)
SMA function, n (%)			
Sitters	31 (68.89)	15 (68.18)	11 (100.00)
Walkers	14 (31.11)	7 (31.82)	0 (0.00)
*SMN2* copies, n (%)			
1	0 (0.00)	0 (0.00)	0 (0.00)
2	3 (8.33)	4 (20.00)	1 (20.00)
3	30 (83.33)	10 (50.00)	3 (60.00)
4	1 (2.78)	1 (5.00)	0 (0.00)
> 4	2 (5.56)	5 (25.00)	1 (20.00)
*Missing*	9 (%)	2 (%)	6 (%)
Duration of treatment at enrolment, n (%)			
≤ 1 year	6 (13.33)	6 (27.27)	–
> 1 year	39 (86.67)	16 (72.73)	–
Oral intake, n (%)			
Solids	43 (95.56)	22 (100.00)	9 (81.82)
Semi-solids	0 (0.00)	0 (0.00)	2 (18.18)
Liquids	0 (0.00)	0 (0.00)	0 (0.00)
Gastrostomy, n (%)	2 (4.44)	0 (0.00)	0 (0.00)

*In non-ambulant patients, recumbent or ulnar length (as per Gauld equation) was used as surrogate for height

All pediatric patients were on steady-state treatment (i.e., after 4 administration of loading dose) with nusinersen. Adult patients on treatment with nusinersen were also on steady-state. Untreated patients are treatment naïve

### Prevalence, Objective and Subjective Features of Oro-bulbar Involvement at Enrolment

#### Paediatric Patients

##### Objective Measures

IOPI

See Table 2.

**Table 2:** Z-scores of the oro-bulbar assessments for ambulant and non-ambulant patients in the paediatric treated cohort

		Overall	Sitters	Walkers	*p*-value*
Tongue strength (*n* = 32)	Median [IQR] *Z*-score	−1.93 [−3.13 to −0.65]	−1.91 [−2.87 to −1.05]	−2.91 [−4.46 to −0.45]	0.5518
	Abnormal, n (%)	15 (46.88)	9 (42.86)	6 (54.55)	0.5291
aMMO (*n* = 44)	Median [IQR] *Z*-score	−1.65 [−3.40 to −0.01]	−2.67 [−4.08 to −0.41]	−0.36 [−1.65 to 0.27]	**0.0185**
	Abnormal, n (%)	19 (43.18)	16 (53.33)	3 (21.43)	0.0466
Number of bites (*n* = 38)	Median [IQR] *Z*-score	−2.82 [−5.63 to −1.00]	−2.79 [−5.45 to −1.00]	−3.34 [−5.63 to −0.76]	0.7581
	Abnormal, n (%)	0 (0.00)	0 (0.00)	0 (0.00)	1.0000
Masticatory cycles (*n* = 38)	Median [IQR] *Z*-score	−0.30 [−5.37 to 3.88]	1.64 [−2.77 to 4.39]	−2.81 [−7.92 to −0.83]	**0.0102**
	Abnormal, n (%)	12 (31.58)	11 (44.00)	1 (7.69)	0.0300
Number of swallows (*n* = 38)	Median [IQR] *Z*-score	4.60 [0.27 to 10.69]	7.14 [0.27 to 10.68]	4.37 [0.27 to 4.94]	0.6222
	Abnormal, n (%)	25 (65.79)	17 (68.00)	8 (61.54)	0.7302
Total time (*n* = 38)	Median [IQR] *Z*-score	1.83 [−2.49 to 5.36]	3.26 [−1.48 to 8.43]	−1.58 [−4.44 to 1.64]	**0.0138**
	Abnormal, n (%)	19 (50.00)	16 (64.00)	3 (23.08)	0.0167

Bold values indicate statistically significant differences

*Z*-scores of < −1.96 (aMMO, IOPI) or >  + 1.96 (TOMASS) were considered abnormal

*p-value refers to the difference between patients functionally sitter and walker to the age and gender-matched normative data

Tongue strength was available in 32 of 45 (71%) children either due to poor collaboration (*n* = 1) or to logistic reasons (*n* = 12).

Tongue strength was below normative data in fifteen of the 32 (47%) patients. Children between 8 and 10 years of age had the highest prevalence of reduced tongue strength [32].

Median *z*-scores < −1.96 for tongue strength at IOPI were considered abnormal. The median tongue strength for the whole paediatric cohort was at the very low end of normal (*z*-score −1.93 [−3.13; −0.65]) without differences between sitters and walkers.

aMMO

The measure of active mouth opening was available in 44 of 45 (98%) paediatric patients. aMMO was missing in one patient for technical reasons.

Overall, 19 of 44 (43%) children had an aMMO below the age-adjusted normative data [33].

Median *z*-scores < −1.96 for aMMO were considered abnormal. The median aMMO for the whole paediatric cohort was at the lower end of normative values (*z*-score −1.65 [−3.40; −0.01]). Children who were functionally classified as sitters had an abnormally reduced mouth opening (*z*-score −2.67 [−4.08; −0.41]. Conversely, children who were functionally classified as walker had a normal mouth opening (*z* score −0.36 [−1.65; 0.27]). This difference was statistically significant (*p* = 0.019).

TOMASS

TOMASS was available in 38 of 45 (84%) paediatric patients, due to either acquisition/reporting difficulties or, in the two patients with gastrostomy, due to a confirmed dysphagia.

Median *z*-scores >  + 1.96 in each of the items tested with TOMASS were considered abnormal. Any item with a median z-score below −1.96 was considered more efficient the more negative its value was.

All children required a normal number of bites to eat a standard cracker.

The median number of masticatory cycles in the paediatric population considered as a whole was normal (*z*-score −0.30 [−5.37; 3.88]). When patients were stratified according to motor function, mastication was found to be more effective in children who were functionally ambulant than in children who were functionally sitters. This difference was statistically significant (*p* = 0.01).

The number of swallows required to eat a standard cracker was abnormally elevated (*z*-score 4.60 [0.27; 10.69]) in both walkers and sitters. Twenty-five of 38 children (66%) had an abnormal number of swallows, most of them being 4 to 6-year-old (*n* = 9) or 8- to 10-year-old (*n* = 6).

The time to eat a cracker and clearing the mouth was at the higher range of normality in the whole paediatric cohort (1.83 [−2.49; 5.36]). Nineteen out of 38 patients (50%) required a prolonged time to eat. Differences were found between sitters and walkers (*p* = 0.014). Patients who were functionally sitters required a prolonged time to eat a standard cracker (*z*-score 3.26 [−1.48; 8.43], whereas walkers required a normal time to eat a cracker (*z*-score −1.58 [−4.44; 1.64]).

The raw values (median and IQR) of oro-bulbar assessments in the paediatric cohort as a whole and stratified by motor functional status are shown in Table 1 supplementary.

##### Subjective Measures

The SMA-HI questionnaire was available in 17 children. Swallowing issues were reported as “burdensome” in 2 of them (12%).

#### Nusinersen Treated Adult Patients

#### Objective Measures

IOPI

See Table 3.

**Table 3:** Z-scores of the oro-bulbar assessments for walkers and sitters in the adult treated cohort

		Overall	Sitters	Walkers	*p*-value*
Lip strength (*n* = 21)	Median [IQR]	−0.17 [−0.91 to 0.94]	−0.41 [−1.00 to 0.80]	1.57 [−0.24 to 2.12]	**0.0321**
	Abnormal, *n* (%)	0 (0.00)	0 (0.00)	0 (0.00)	1.0000
Tongue strength (*n* = 21)	Median [IQR]	−1.22 [−2.29 to −0.58]	−1.21 [-2.29 to −0.58]	−1.09 [−3.10 to 0.09]	0.9992
	Abnormal, *n* (%)	7 (33.33)	5 (33.33)	2 (33.33)	1.0000
aMMO (*n* = 21)	Median [IQR]	−1.40 [−2.59 to −0.01]	−1.75 [−3.89 to −0.15]	−0.56 [−1.68 to 0.73]	0.0931
	Abnormal, *n* (%)	8 (38.10)	7 (50.00)	1 (14.29)	0.1736
Number of bites (*n* = 21)	Median [IQR]	−4.63 [−4.63 to −3.33]	−4.63 [−4.63 to −3.33]	−4.55 [-4.63 to −1.12]	0.4136
	Abnormal, *n* (%)	0 (0.00)	0 (0.00)	0 (0.00)	1.0000
Masticatory cycles (*n* = 21)	Median [IQR]	−4.48 [−6.52 to −2.38]	−4.54 [−6.52 to −1.41]	−4.48 [−6.86 to −2.40]	0.7393
	Abnormal, *n* (%)	2 (9.52)	2 (14.29)	0 (0.00)	0.5333
Number of swallows (*n* = 21)	Median [IQR]	−0.94 [−4.05 to 5.31]	−2.21 [−5.00 to 5.48]	−0.94 [-2.41 to 5.31]	0.6798
	Abnormal, *n* (%)	9 (42.86)	6 (42.86)	3 (42.86)	1.0000
Total time (*n* = 21)	Median [IQR]	−1.32 [−4.45 to 5.02]	−0.59 [−3.63 to 10.02]	−3.43 [−5.66 to −0.81]	0.1457
	Abnormal, *n* (%)	6 (28.57)	5 (35.71)	1 (14.29)	0.6126

Bold values indicate statistically significant differences

*Z*-scores of < −1.96 (aMMO, IOPI) or >  + 1.96 (TOMASS) were considered abnormal

*p value refers to the difference between patients functionally sitter and walker to the age and gender-matched normative data

Median *z*-scores < −1.96 for lip and tongue strength at IOPI were considered abnormal.

Lip strength was normal in all the 21 patients tested out of 22. Differences were found between walkers and sitters (*p* = 0.032).

The median value of tongue strength was within normal range (*z*-score −1.22 [−2.29; −0.58]) in the adult treated population. Seven out of the 21 (33%) patients had a reduced tongue strength. No differences were found between patients characterized as sitters and walkers.

aMMO

The measure of the aMMO was available in 21 patients; 8 of them (38%) had a reduced mouth opening.

Median z-scores < −1.96 for active mouth opening were considered abnormal.

The median value was within normal range (*z*-score −1.40 [−2.59; −0.01]) in the whole cohort as well as into both subgroups of sitters and walkers.

TOMASS

Median *z*-scores >  + 1.96 in each of the items tested at TOMASS were considered abnormal.

The number of bites required to eat a cracker was within the range of normality in all 21 tested patients.

The mastication efficiency was clinically normal (*z*-score −4.48 [−6.52; −2.38]) in the overall population and in both motor functional subgroup. The number of masticatory cycles was higher than normative data in 2 out of 21 patients (10%).

The median number of swallows was within normal range (*z*-score −0.94 [−4.05; 5.31]) in the overall cohort and in patients who were functionally characterised as walkers and sitters. The number of swallows was elevated in 9 of 21 patients (43%).

The overall median time required to eat a standard cracker was within normal range in the adult treated cohort (*z*-score −1.32 [−4.45; 5.02]). Notably, patients who were functionally walkers had a shorter median time than normative data (*z*-score −3.43 [−5.66; −0.81]), suggesting preserved mastication and swallowing abilities. The total time to eat was prolonged in 6 of 21 patients (29%).

##### Subjective Measures

SMA-HI was available for 21 out of the 22 patients. The results of the perceived burden in the oro-bulbar functions in adult patients differed depending on whether they were on regular treatment or not. Five of 21 treated patients (24%) reported any issues with swallowing (i.e., SMA-HI swallowing above 0).

#### Untreated Adult Patients

##### Objective Measures

IOPI

See Table 4.

**Table 4:** Z-scores of the oro-bulbar assessments for sitters in the adult untreated cohort

		Overall
Lip strength (*n* = 9)	Median [IQR]	−0.80 [−1.00 to −0.66]
	Abnormal, *n* (%)	0 (0.00)
Tongue strength (*n* = 8)	Median [IQR]	−2.20 [−3.34 to −0.94]
	Abnormal, *n* (%)	4 (50.00)
aMMO (*n* = 10)	Median [IQR]	−2.68 [−3.75 to −1.87]
	Abnormal, *n* (%)	7 (70.00)
Number of bites (*n* = 8)	Median [IQR]	−4.63 [−7.42 to −3.98]
	Abnormal, *n* (%)	0 (0.00)
Masticatory cycles (*n* = 8)	Median [IQR]	−1.64 [−7.26 to 3.54]
	Abnormal, *n* (%)	4 (50.00)
Number of swallows (*n* = 8)	Median [IQR]	1.19 [−4.06 to 11.77]
	Abnormal, *n* (%)	3 (37.50)
Total time (*n* = 8)	Median [IQR]	−0.59 [−4.06 to 8.36]
	Abnormal, *n* (%)	3 (37.50)

*Z*-scores of < −1.96 (aMMO, IOPI) or >  + 1.96 (TOMASS) were considered abnormal

Lip strength was normal in the 9 patients who had this ability tested.

The median tongue strength was reduced compared to the normative data (*z*-score −2.20 [−3.34 to −0.94]) with 4 of 8 patients having a reduced strength (50%).

aMMO

AMMO was available in ten patients; seven of them (70%) had a reduced mouth opening. The median mouth opening was lower than normative data (*z*-score −2.68 [−3.75 to −1.87]).

TOMASS

The number of bites was within the range of normality in all eight patients tested.

The median number of masticatory cycles was at the lower end of normality (*z*-score −1.64 [−7.26 to 3.54]). Four out of eight patients (50%) had an increased number of masticatory cycles.

The median number of swallows to eat the cracker and clearing the mouth was similar to normative adults (*z*-score 1.19 [−4.06 to 11.77]). Three of 8 patients had an increased number of swallows (38%).

Overall, the median total time taken to eat a cracker was similar to healthy adults (*z*-score −0.59 [−4.06 to 8.36]). The time to eat the cracker was prolonged in 3 out of 8 patients (38%).

The raw values (median and IQR) of oro-bulbar assessments in untreated and treated adults are shown in supplementary Tables 2 and 3.

#### Subjective Measures

SMA-HI was available in 5 out of 11 patients. All 5 patients reported swallowing to be a burden.

### Correlation Between Active Maximal Mouth Opening, Lip and Tongue Strength and the Time to Eat (Using TOMASS)

Univariate regression analysis showed that aMMO significantly contributed to the time needed to finish a standardized cracker in the overall study population (*F* = 13.88, *p* = 0.0005), both in the paediatric (*F* = 20.09, *p* = 0.0001) and in the treated adult (*F* = 13.16, *p* = 0.0018) cohorts. No significant contribution of mouth opening to the time to eat a cracker was found in the adult untreated group.

More in detail, the mean effect of an increase of 1 mm in aMMO corresponded to a reduction in time needed to finish the cracker of 2.0% (95% CI 0.93 to 3.07) in the overall population, 3.3% (95% CI 1.81 to 4.87) in the paediatric cohort and 2.7% (95% CI 1.14 to 4.20) in the adult treated group.

Lip strength assessed via IOPI did not correlate with the time to eat using/assessed by at TOMASS in any of the study cohorts. Conversely, tongue strength significantly contributed to both a shorter time to eat (*F* = 6.42, *p* = 0.0142) and reduced swallowing cycles (*F* = 5.64, *p* = 0.0212).

In the pediatric cohort, for each 1 kPa increase in tongue strength, there was a 0.9% reduction in the total time to eat a cracker (95% CI 0.18 to 1.57) and a 0.8% reduction in the number of swallowing cycles (95% CI 0.12 to 1.55). No significant relationship emerged in both adult treated and untreated groups, separately.

### Progression of Swallowing and Mastication Over Time

The progression of swallowing and mastication was evaluated every 4 months at each nusinersen administration for up to 16 months (median f-u of 8 months [4–12]). Since all patients who were untreated at enrolment started a disease modifying treatment after the first visit, they were excluded from the longitudinal analysis.

#### IOPI

The median tongue strength remained below normative data in the paediatric cohort ranging from *z*-score −2.12 [−3.04; −1.22] at baseline (*n* = 20 pts) to a *z*-score −2.21 [−2.87; −0.36] at month 16 (*n* = 6 pts) and within normal values in the adult cohort, ranging from *z*-score −1.23 [−2.29; −0.61] at baseline (*n* = 16 pts) to z-score −0.39 [−1.54; 0.32] at month 16 (*n* = 6 pts) without significant differences over time. Adults also maintained normal lip strength throughout the follow-up ranging from a *z*-score −0.33 [−0.91; 0.83] at baseline (*n* = 16) to a *z*-score −0.81 [−1.49; −0.33] at month 16 (*n* = 6 pts).

#### aMMO

The mouth opening progressively reduced over time in the paediatric population from values within normal range at baseline (*z*-score −1.65 [−3.07; 0.13], in 34 patients) to values below normative range at the latest follow-up (*z*-score −2.22 [−3.24; −0.63]) at month 16 (in six patients). This difference, however, was not significant.

Mouth opening remained within normal range over time in the adult population (from *z*-score −1.17 [−3.47; −0.01] at baseline, in 18 patients; to *z*-score −1.04 [−2.91; −0.74] at month 16, in six patients).

#### TOMASS

In the paediatric cohort, none of the TOMASS’ derived items did significantly change during the observation period. However, the number of swallows changed from being elevated (*z*-score 4.44 [0.00; 10.27] at baseline (in 25 patients) to normal (*z*-score −1.41 [−2.14; 3.52]) at month 16 (in three patients).

**Fig. 2:**
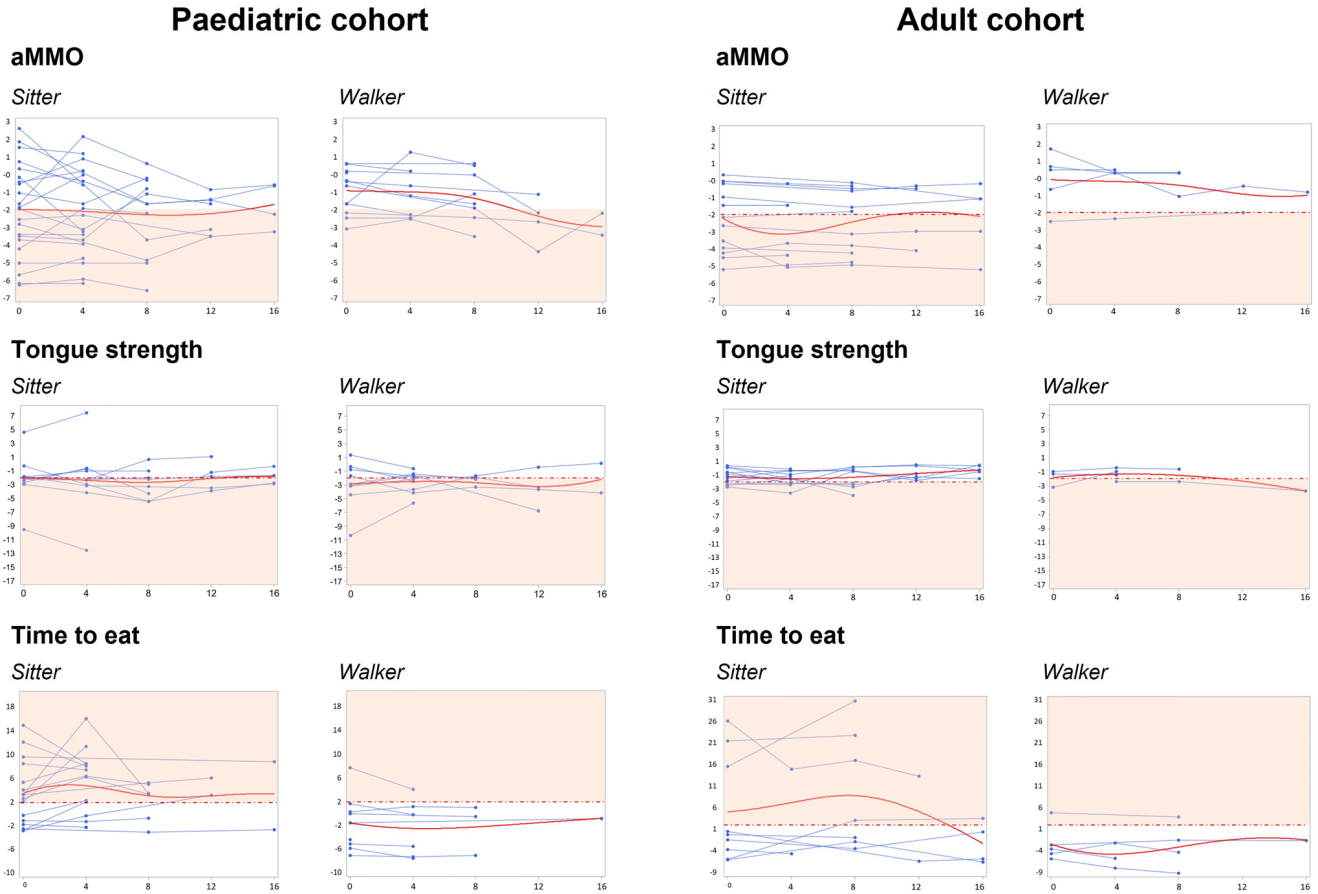
Progression of swallowing and mastication in children and adults treated with nusinersen. Data are expressed as Z-score (y axis) and months of follow-up (x axis). Any value below (for aMMO and tongue strength) or above (for time to eat) 1.96 SD (dotted red line) was considered pathological (red filled area)

Similarly, there was no difference in mastication and swallowing over time in adults; however, the number of swallows overall improved, starting from being normal (*z*-score −1.67 [−4.05; 5.48] at baseline (in 14 patients) to being reduced (*z*-score −2.41 [−3.47; −1.67]) at month 16 (in five patients). (Fig. 2).

No difference was observed in the decline of any of the variables assessed when children and adults were stratified according to the duration of nusinersen (shorter or longer than 1 year).

## Discussion

In this study a combined set of tools was used to assess oro-bulbar function in patients with SMA type 2 and 3. Mouth opening and tongue strength were mostly affected in SMA types 2 and 3 and especially in children and in sitters compared to walkers. Additionally, the results confirm a discrepancy between the subjective perception of impaired mastication and swallowing reported by patients and the objective measurements of mouth opening, lip and tongue strength and mastication and swallowing. Finally, mouth opening, tongue strength and time to eat a cracker were found to be stable over time in nusinersen treated patients.

Oro-bulbar issues were found to be highly prevalent in patients with SMA in this study. They result from the combination of the mechanical limitations of mouth opening and the neurological dysfunction affecting jaw, tongue and facial muscles. [10] This study also demonstrated that, in patients with SMA, each of these aspects contribute differently to the total time taken to clear the mouth and this varies according to age groups and motor functional status.

The strength generated by lip and tongue was assessed for the first time in patients with SMA in this study using the Iowa Oral Performance Instrument and was compared to age-matched normative data.

Almost 50% of children on steady nusinersen were found to have a weak tongue. There was no difference in tongue strength between walkers and sitters. Conversely, in adults with SMA on steady treatment, both ambulant and non-ambulant, lip and tongue do not seem to be weak. All 11 untreated adult patients, all sitters, had tongue strength reduced.

The active maximum mouth opening was measured and was found to be impaired, being at the lower end of normality, in both children and adults with SMA. Up to 43% (19 of 44) children had a reduced opening. Notably, ambulant children had a significantly wider jaw motion (*p* = 0.019) than non-ambulant. Amongst adults, 33% (8 of 21) treated and all 11 (100%) untreated, all sitters, had a reduced mouth opening.

In this study, mastication and swallowing in the paediatric and adult cohorts were also assessed via TOMASS. As found in other studies, whilst treated adult (both sitters and walkers) had swallows and mastication abilities within normative range, there were differences between sitters and walkers only in the pediatric cohort. The sub-group of ambulant children chew as effectively as healthy children (less cycle required, z score—2.8) and significantly better than sitters (*p* = 0.01). Similarly, the time to eat was prolonged only in children who were sitters (z-score 3.3) and was significantly longer than in walkers (*p* = 0.014). The only available study evaluating TOMASS in a cohort of children and adults with SMA type 2 and 3, showed that masticatory cycles and swallows were affected only in sitters [15].

The three sets of tests allowed also to confirm the actual interplay between tongue strength, mouth opening and bolus processing to the oral phase of swallowing.

A limited mouth opening, in combination with mastication problems, is one the main feature in SMA type 2 and 3 [17–19, 39]. A limited mouth opening, in combination with mastication problems, is one the main feature in SMA type 2 and 3. Active mouth opening is related to the action of the pterygoid muscles and submental muscle group, which are known to be affected by the disease [10, 14, 19, 40]. These are innervated by the 5th and 7th cranial nerves and therefore also related to brainstem functions. A wider aMMO positively affects the swallowing assessed via TOMASS in a cohort of 27 children and adults with SMA [15]. Interestingly, this study further adds that the reduction of the time to eat a cracker (assessed by the TOMASS) for each extra 1 mm aMMO accounts by 3.3% in children, 2.7% in treated adults but was not found in the untreated adult cohort. This may suggest that mandibular rehabilitation deserves more attention in patients living with SMA and could potentially amplify or coadjuvate the effects of pharmacological treatment.

Furthermore, the tongue plays an important role in initiating the oral phase of swallowing and in the propulsion of the bolus within the mouth. We can speculate that a reduction in the number of swallowing cycles and a shorter time to eat a cracker based on the TOMASS scores is related to a greater strength in the tongue muscles. In this study, tongue strength significantly contributed to both a shorter time to eat (*F* = 6.42, *p* = 0.0142) and reduced swallowing cycles (*F* = 5.64, *p* = 0.0212).

Swallowing is composed by three stages: oral, pharyngeal and oesophageal. In SMA type 2 and 3 the main features of dysphagia involve the oro-pharyngeal phases of swallowing, resulting in pyriform and vallecular residue requiring repeated swallowing acts [41] [42]. The aforementioned deficits in chewing and swallowing solids have been linked to impairments in mandibular range of motion and strength [14, 19, 40]. Muscle contracture of the temporomandibular joint characterized by restriction in jaw range of motion was found to be correlated with SMA type [40]. This was also observed in the paediatric cohort of this study, where sitters had a reduced aMMO than walkers, but no differences were found amongst adults.

An impaired swallow defined by the presence of aspiration and penetration has lower prevalence than oro-pharyngeal issues in SMA type 2 and 3 [41] [10]. Chacko et al. recently found that around 30% pts with SMA type 2 and 3 have dysphagia at VFSS [43]. It may possibly occur at a stage when patients experience also significant respiratory impairment and fatigue. The assessment of oro-pharyngeal impairment via clinical tests is crucial at this stage and particularly in treated patients as it can be used to stratify the risk of swallowing as a whole and direct clinicians towards the need of video fluoroscopy (VFSS).

This study also compared the objective measures of oro-bulbar function with patients’ subjective perception. Interestingly, despite the high prevalence of objective impairment, only a minority of treated adult and children in this study (24 and 12%, respectively) complained of mastication and swallowing issues defined by an SMA-HI score greater than 0. Conversely, 5 of 5 untreated adults reported any subjective concern with swallowing, supporting the role of treatment also on the perception of the disease-related aspects.

In previous studies, 30% of patients reported limitations with jaw opening, 26% issues with chewing, 20.4% difficulty conveying food to the mouth and 30.6% choking [9, 13, 39]. Swallowing issues and choking were rated as impacting 2 in a disease burden scale 0–4 in 359 adult patients with SMA in the PRISM study [11].

It is worth noting that in this study only 12% treated children perceived burden in mastication or swallowing, yet 29% had a BMI below normal range. Specific growth chart for SMA type 1 and 2 have been created [44], however they were not used for comparison in this study as they do not include ambulant patients. The impairment in oropharyngeal abilities and craniofacial morphology often result in weight loss and need for gastrostomy. In a cohort of 142 untreated paediatric patients with SMA type 2 from Italy and UK, 60% had periods of underweight and 25% were chronically malnourished. Enteral feeding was indicated in 32% UK patients [16]. In this study only 4% of the treated children had required an adapted diet or a gastrostomy.

Finally, whilst clinical trials [45, 46], and real-world data [47–50], reported beneficial role of disease modifying treatments in motor and respiratory function, data on the impact of treatment on mastication and swallowing in SMA type 2 and 3 is scanty. In adult patients with SMA type 2 and 3, age has proven to be a significant determinant of facial muscle impairment, suggesting a trend towards the progression of bulbar involvement over time. [14] The longitudinal exploratory data in the treated paediatric and adult cohorts in this study suggests that nusinersen may stabilize the oro-pharyngeal abilities after an average follow-up of 8 months. No significant differences in the progression of oro-bulbar assessments in adults and children were found when comparing first and latest assessments. Whilst adults with SMA had no changes in any of the function tested, children had a trend towards reduced swallowing cycles, them ranging from abnormally high at first assessment to normal at latest observation. No rehabilitation was performed on jaw and facial muscles. Therefore, we cannot exclude the effects of pharmacological treatment, although this interpretation should be taken with caution and further data are needed as the lack of natural history data limit any further conclusion.

The widespread availability of disease modifying treatments for paediatric and adult patients with Spinal Muscular Atrophy highlighted the multifaceted aspects of the disease. This is, to our knowledge, the first report of the oro-bulbar involvement in adults and paediatric patients with SMA type 2 and 3 either untreated or during steady treatment with nusinersen. In this study a set of validated and exploratory tests was conducted by experienced professionals in two tertiary care centres highly specialized in SMA. TOMASS and mouth opening were conducted in keeping with previous publications. [15] The assessment of tongue and lip strength via IOPI is novel in SMA and has proven to complement well the other tests in both adults and children.

Despite these encouraging considerations, there are major limitations to this study, the most important being the absence of a paediatric untreated group and the absence of natural history data on mastication and swallowing functions in patients with SMA. When the study was designed the only available treatment for SMA types 2 and 3 was nusinersen and all children and many adults were already treated. As a consequence, patients on treatment were included at different timepoints of their treatment schedule. To reduce variability, only patients on steady nusinersen regime were included. Given the considerations above, a formal comparison of oro-bulbar function between treated and untreated adult patients was beyond the scope of the study and was, therefore, not performed. Oro-bulbar outcomes were presented separately in the two cohorts and matched analyses were not conducted in this study.

The additional limitations are intrinsic to the tests rather than subsequent to their application in patients with SMA. The assessment of lip and tongue strength and aMMO, but not TOMASS, require the subjects’ maximum performance. They are therefore affected by the comprehension of the task, motivation and by the effect of fatigue. These aspects potentially account for different levels of performance even within the same subject. To reduce the risk of patients performing below maximum during mouth opening, the evaluators prompted the patient to perform as best and observed masseter muscle contraction. During the IOPI performances the best of three efforts was selected. In addition, all patients were cognitively unaffected and very compliant.

Although the frontal incisor distance between gums would potentially affect mouth opening in children, the age range of the children on treatment were compared to age-matched children from normative data. Dentition did not seem to account for the trend (although not significant) of a steady mouth opening in treated patients over time. The presence of high arched palate in patients with SMA can potentially affect the measurement of tongue strength and should be recorded at the time of assessment.

Additionally, whilst undoubtedly fatigability affects mastication and swallowing in SMA, this study was designed to identify tools for the measurement of oro-bulbar function only.

In conclusion, oro-bulbar issues are prevalent in both children and adults living with SMA and should be assessed despite patients’ poor perception of symptoms. As mouth opening and tongue strength are crucial to the swallowing process, a multimodal set of assessments may be used as first-line approach to determine whether more invasive evaluations are needed. Further studies are required to identify which of the tools described best capture changes with treatment, either pharmacological or non-pharmacological. Finally, the longitudinal data showing the stability of oro-bulbar abilities in the treated cohorts are encouraging but longer follow-up on broader cohorts are needed.

### Supplementary Information

Below is the link to the electronic supplementary material.

Supplementary file1 (DOCX 14 KB)

Supplementary file2 (DOCX 15 KB)

Supplementary file3 (DOCX 13 KB)
