# Supplementary material for: Assessing Prevalence and Characteristics of Oro-bulbar Involvement in Children and Adults with SMA Type 2 and 3 Using a Multimodal Approach
Source: Dysphagia. 2023 Jun 8;38(6):1568–80. doi: 10.1007/s00455-023-10584-z (PMC10611831; doi:10.1007/s00455-023-10584-z)
Supplement: Supplementary file 1 — Supplementary file1 (DOCX 14 KB) [file 455_2023_10584_MOESM1_ESM.docx]

|  | **Overall paediatric treated cohort (n=45)** | | |
| --- | --- | --- | --- |
| **BMI**, median [IQR], kg/m2 | 15.67 [13.52 – 19.05] (n=44) | | |
| **AMMO**, median [IQR],mm | 37.50 [30.50 – 44.50] (n=44) | | |
| **Lip strength**, median [IQR],kPa | 18.50 [10.50 – 27.00] (n=32) | | |
| **Tongue strength**, median [IQR], kPa | 22.50 [14.50 – 35.50] (n=32) | | |
| **Number of bites**, median [IQR],n | 2.50 [2.00 – 4.00] (n=38) | | |
| **Masticatory cycles**, median [IQR],n | 47.00 [36.00 – 63.00] (n=38) | | |
| **Number of swallows**, median [IQR], n | 3.50 [2.00 – 5.00] (n=38) | | |
| **Total time**, median [IQR], sec | 47.72 [34.47 – 66.00] (n=38) | | |
|  |  | | |
|  | **Sitters (n=31)** | **Walkers (n=14)** | ***p-value*** |
| **BMI**, median [IQR], kg/m2 | 15.29 [13.22 – 19.70] | 16.00 [14.35 – 17.70] | *0.7913* |
| **AMMO**, median [IQR],mm | 35.00 [28.00 – 43.00] | 44.00 [35.00 – 46.00] | ***0.0171*** |
| **Lip strength**, median [IQR],kPa | 23.00 [7.00 – 28.00] | 14.00 [13.00 – 24.00] | *0.9683* |
| **Tongue strength**, median [IQR], kPa | 24.00 [15.00 – 35.00] | 21.00 [14.00 – 41.00] | *0.9997* |
| **Number of bites**, median [IQR],n | 2.00 [2.00 – 4.00] | 3.00 [2.00 – 4.00] | *0.7532* |
| **Masticatory cycles**, median [IQR],n | 54.00 [42.00 – 73.00] | 38.00 [23.00 – 44.00] | ***0.0201*** |
| **Number of swallows**, median [IQR], n | 4.00 [2.00 – 5.00] | 3.00 [3.00 – 4.00] | *0.4705* |
| **Total time**, median [IQR], sec | 57.52 [43.25 – 66.03] | 41.41 [32.00 – 53.02] | ***0.0472*** |

**Table 1 supplementary –** Oro-bulbar assessments in paediatric treated cohort
