# Supplementary material for: Assessing Prevalence and Characteristics of Oro-bulbar Involvement in Children and Adults with SMA Type 2 and 3 Using a Multimodal Approach
Source: Dysphagia. 2023 Jun 8;38(6):1568–80. doi: 10.1007/s00455-023-10584-z (PMC10611831; doi:10.1007/s00455-023-10584-z)
Supplement: Supplementary file 2 — Supplementary file2 (DOCX 15 KB) [file 455_2023_10584_MOESM2_ESM.docx]

|  | **Overall adult treated cohort (n=22)** | | |
| --- | --- | --- | --- |
| **BMI**, median [IQR], kg/m2 | 22.88 [20.75 – 26.22] (n=22) | | |
| **AMMO**, median [IQR],mm | 42.00 [36.00 – 52.00] (n=21) | | |
| **Lip strength**, median [IQR],kPa | 25.00 [17.00 – 41.00] (n=21) | | |
| **Tongue strength**, median [IQR], kPa | 39.00 [30.00 – 48.00] (n=21) | | |
| **Number of bites**, median [IQR],n | 1.00 [1.00 – 2.00] (n=21) | | |
| **Masticatory cycles**, median [IQR],n | 30.00 [25.00 – 35.00] (n=21) | | |
| **Number of swallows**, median [IQR], n | 2.00 [2.00 – 3.00] (n=21) | | |
| **Total time**, median [IQR], sec | 26.34 [21.79 – 53.30] (n=21) | | |
|  |  | | |
|  | **Sitters (n=15)** | **Walkers (n=7)** | ***p-value*** |
| **BMI**, median [IQR], kg/m2 | 23.36 [21.48 – 26.22] | 22.39 [15.87 – 26.40] | *0.4809* |
| **AMMO**, median [IQR],mm | 41.00 [24.00 – 51.00] | 48.00 [40.00 – 56.00] | *0.1083* |
| **Lip strength**, median [IQR],kPa | 22.00 [16.00 – 39.00] | 48.50 [24.00 – 58.00] | ***0.0291*** |
| **Tongue strength**, median [IQR], kPa | 43.00 [30.00 – 48.00] | 38.00 [14.00 – 57.00] | *0.9689* |
| **Number of bites**, median [IQR],n | 1.00 [1.00 – 2.00] | 2.00 [1.00 – 2.00] | *0.3144* |
| **Masticatory cycles**, median [IQR],n | 31.00 [23.00 – 35.00] | 28.00 [25.00 – 35.00] | *0.9107* |
| **Number of swallows**, median [IQR], n | 2.00 [2.00 – 4.00] | 2.00 [2.00 – 3.00] | *0.9066* |
| **Total time**, median [IQR], sec | 34.00 [24.00 – 55.00] | 23.13 [20.00 – 30.52] | *0.2475* |

**Table 2 supplementary –** Oro-bulbar assessments in adult treated cohort (n=22)
