# Supplementary material for: Assessing Prevalence and Characteristics of Oro-bulbar Involvement in Children and Adults with SMA Type 2 and 3 Using a Multimodal Approach
Source: Dysphagia. 2023 Jun 8;38(6):1568–80. doi: 10.1007/s00455-023-10584-z (PMC10611831; doi:10.1007/s00455-023-10584-z)
Supplement: Supplementary file 3 — Supplementary file3 (DOCX 13 KB) [file 455_2023_10584_MOESM3_ESM.docx]

|  | **Overall adult naive cohort (n=11)** |
| --- | --- |
| **BMI**, median [IQR], kg/m2 | 22.23 [21.73 – 27.03] (n=7) |
| **AMMO**, median [IQR],mm | 32.50 [25.00 – 37.00] (n=10) |
| **Lip strength**, median [IQR],kPa | 17.00 [14.00 – 19.00] (n=9) |
| **Tongue strength**, median [IQR], kPa | 28.00 [14.50 – 44.00] (n=8) |
| **Number of bites**, median [IQR],n | 1.00 [1.00 – 1.00] (n=8) |
| **Masticatory cycles**, median [IQR],n | 32.50 [21.00 – 55.00] (n=8) |
| **Number of swallows**, median [IQR], n | 3.00 [1.50 – 5.00] (n=8) |
| **Total time**, median [IQR], sec | 27.11 [23.86 – 57.19] (n=8) |

**Table 3 supplementary –** Oro-bulbar assessments in adult naive cohort
